# Authors’ Reply to: To Screen or Not to Screen? At Which BMI Cut Point? Comment on “Obesity and BMI Cut Points for Associated Comorbidities: Electronic Health Record Study”

**DOI:** 10.2196/39717

**Published:** 2022-06-29

**Authors:** Luke Funk, Natalie Liu

**Affiliations:** 1 Department of Surgery University of Wisconsin-Madison Madison, WI United States; 2 Department of Surgery William S Middleton VA Madison, WI United States

**Keywords:** obesity, body mass index, BMI, risk factors, screening, health services, chronic disease, heart disease, myocardial perfusion imaging, anxiety, depression

We thank our colleague from Greece [1] for her interest in our article [2]. Similar to our study, Fotopoulos et al [3] found an association between obesity and coronary artery disease (CAD) in their analysis of patients undergoing cardiac stress tests. They also reported that the presence of obesity and depression together was associated with CAD. Similarly, the presence of obesity and anxiety together was associated with CAD. Our study did not explicitly measure associations between depression and CAD or anxiety and CAD, but Sioka [1] raised important points about how obesity, mental health, and heart disease may interact.

In the adjusted quantile regression analyses (Multimedia Appendix 4 of our paper), we found that patients with a diagnosis of anxiety had a similar BMI as those without anxiety. One systematic review of the literature suggested a positive association between obesity and anxiety, although a causal relationship has not been established [4]. Patients in our study who had a diagnosis of depression had a slightly higher median BMI than those without depression (0.74 BMI points, 95% CI 0.53-0.94). A meta-analysis of 15 longitudinal studies concluded that obesity increased the risk of depression, and depression was predictive of developing obesity [5].

Our study [2] and the study by Fotopoulos and colleagues [3] both reinforce the concept that obesity is associated with negative health outcomes that affect numerous body systems. Incorporating BMI into screening guidelines for conditions like CAD may help identify high-risk individuals so they can be intervened on earlier than current guidelines support.

## Abbreviations

CAD: coronary artery disease
